# SEX DIFFERENCES IN EMPLOYMENT HISTORY AND LATE-LIFE COGNITION AMONG OLDER CHINESE

**DOI:** 10.1093/geroni/igad104.3589

**Published:** 2023-12-21

**Authors:** Peiyi Lu, Hongyu Yin, Chihua Li

**Affiliations:** Columbia University, New York City, New York, United States; University of California Irvine, Irvine, Irvine, California, United States; University of Michigan, Ann Arbor, Michigan, United States

## Abstract

This study characterizes the employment history of older Chinese men and women and examines the associations between employment trajectories and late-life cognition. Individuals aged 60+ in the 2011 China Health and Retirement Longitudinal Study were included (2,733 men and 2,363 women). Multiple waves of surveys were conducted to assess their cognition in 2011, 2013, and 2015. Sequence analysis identified employment trajectories among men and women separately based on self-reported employment history of whole working age (15-60 years). Mixed-effect models examined the relationship between employment trajectories and cognition. Three distinctive employment trajectories were identified for men and women, separately. Among men, 45% men was consistently agriculture employed, 26% were consistently non-agriculture employed and retired at age 60, and 29% had fluctuating employment featuring in agriculture and early-retirement. Among women, 70% women was consistently agriculture employed, 15% were consistently non-agriculture employed and retired at age 55, and 14% had fluctuating employment featuring unemployment, home, and early-retirement. Compared to the consistently agriculture employed, the consistently non-agriculture employed group reported better cognition for both men and women (men: β=1.26, 95%CI=0.81, 1.66; women: β=1.63, 95%CI=1.09, 2.30). Men with fluctuating employment trajectory also had 0.62 points higher cognition (95%CI=0.27, 0.96) than men who were consistently agriculture employed. Older Chinese experienced distinct life-course employment trajectories reflecting the economic and historic development in China. The sex difference was evident as women were more involved in agricultural work and working at home. Non-agricultural employment was associated with better cognition, highlighting the disadvantaged health status of Chinese farmers.

